# Common neuropathological features underlie distinct clinical presentations in three siblings with hereditary diffuse leukoencephalopathy with spheroids caused by *CSF1R* p.Arg782His

**DOI:** 10.1186/s40478-015-0219-x

**Published:** 2015-07-04

**Authors:** John L. Robinson, EunRan Suh, Elisabeth M. Wood, Edward B. Lee, H. Branch Coslett, Kevin Raible, Virginia M.-Y. Lee, John Q. Trojanowski, Vivianna M. Van Deerlin

**Affiliations:** Department of Pathology and Laboratory Medicine, Center for Neurodegenerative Disease Research, Perelman School of Medicine at the University of Pennsylvania, 3600 Spruce Street, 19104 Philadelphia, PA USA; Department of Neurology, Perelman School of Medicine at the University of Pennsylvania, Philadelphia, PA USA

**Keywords:** Leukoencephalopathy, Microglia, HDLS, *CSF1R*, Frontotemporal degeneration, Corticobasal syndrome, Dementia with Lewy bodies

## Abstract

Hereditary diffuse leukoencephalopathy with spheroids (HDLS) presents with a variety of clinical phenotypes including motor impairments such as gait dysfunction, rigidity, tremor and bradykinesia as well as cognitive deficits including personality changes and dementia. In recent years, colony stimulating factor 1 receptor gene (*CSF1R*) has been identified as the primary genetic cause of HDLS. We describe the clinical and neuropathological features in three siblings with HDLS and the *CSF1R* p.Arg782His (c.2345G > A) pathogenic mutation. Each case had varied motor symptoms and clinical features, but all included slowed movements, poor balance, memory impairment and frontal deficits. Neuroimaging with magnetic resonance imaging revealed atrophy and increased signal in the deep white matter. Abundant white matter spheroids and CD68-positive macrophages were the predominant pathologies in these cases. Similar to other cases reported in the literature, the three cases described here had varied clinical phenotypes with a pronounced, but heterogeneous distribution of axonal spheroids and distinct microglia morphology. Our findings underscore the critical importance of genetic testing for establishing a clinical and pathological diagnosis of HDLS.

## Background

Hereditary diffuse leukoencephalopathy with spheroids (HDLS) is a rare, autosomal-dominant neurodegenerative disease that presents with diverse phenotypes including motor impairments such as gait dysfunction, rigidity, tremor and bradykinesia along with cognitive impairments like personality changes and dementia [1]. The onset of symptoms is usually in the fourth or fifth decade, progressing to dementia and death within 5–10 years. Magnetic resonance imaging (MRI) typically shows patchy cerebral white matter abnormalities [2]. A definite diagnosis of HDLS requires pathology demonstrating widespread myelin loss and abundant axonal spheroids. Since the discovery of mutations in the colony stimulating factor 1 receptor gene (*CSF1R*) that are pathogenic for HDLS [3], genetic screening of *CSF1R* has increased the number of individuals diagnosed with HDLS [4–9]. Here we describe the clinical and neuropathological features of three siblings with a previously published pathogenic *CSF1R* mutation, p.Arg782His [1].

### Genetics

**Fig. 1:**
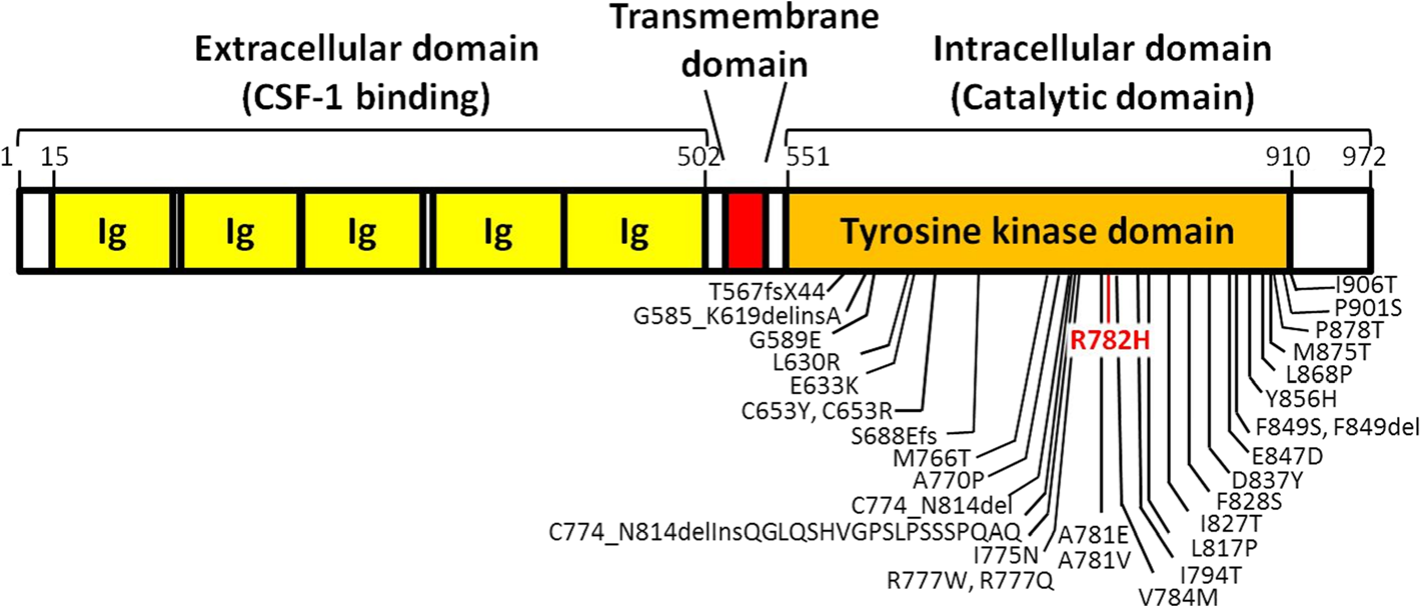
*CSF1R* protein domain and mutation schematic. Schematic diagram of the protein domain structure of *CSF1R* with amino acid numbers provided. Mutations previously reported in other studies are shown in black [3–5, 7–9] and the R782H mutation identified in the present study is highlighted in red. Ig: Immunoglobulin domains

**Fig. 2:**
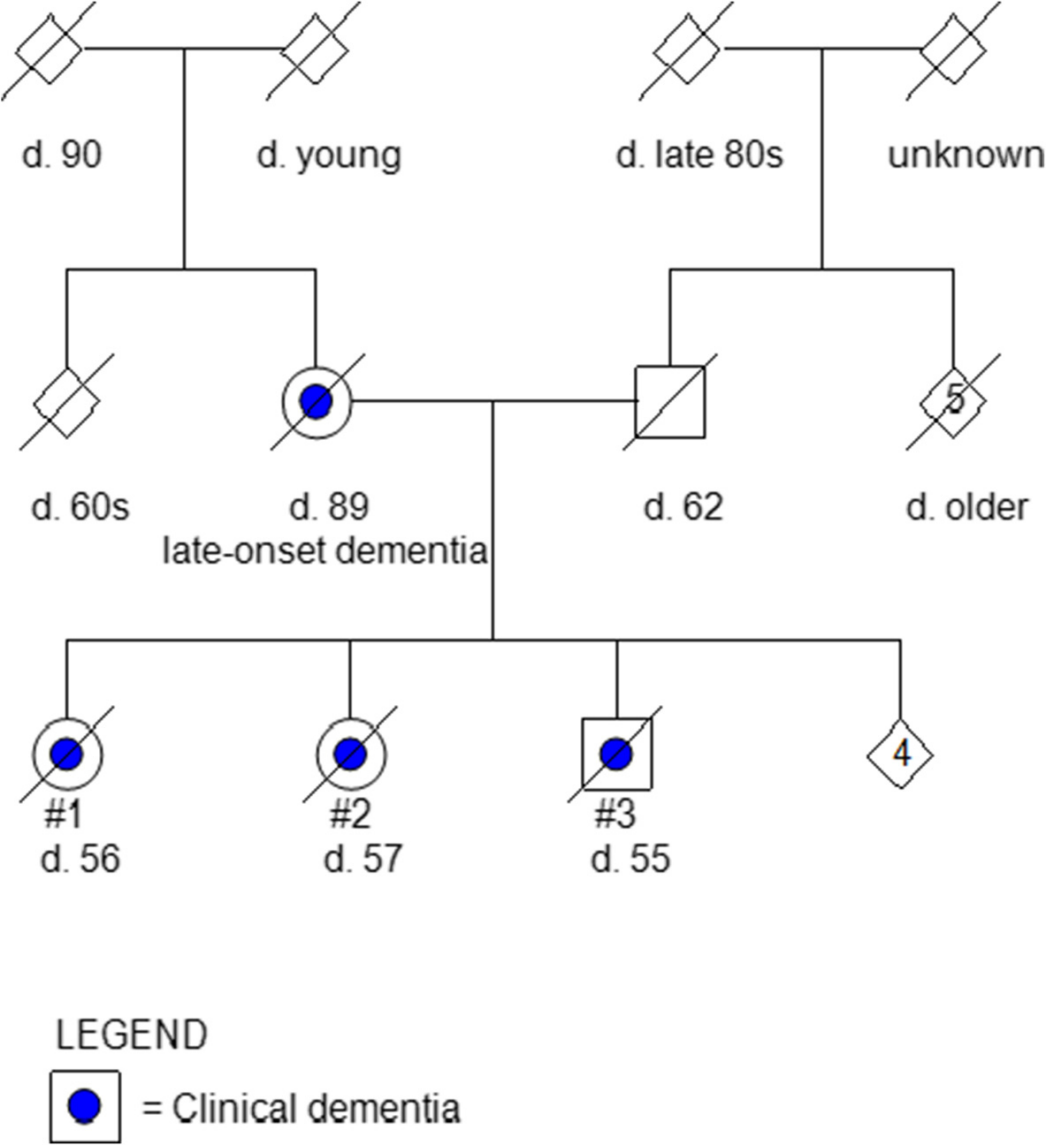
Family Pedigree. A three-generation pedigree of the family is shown. The symbols for individuals symptomatic with any form of dementia are filled with blue circles. Deceased individuals have a slash mark with age at death (d.) indicated. Diamond shapes are intended to mask gender for privacy protection. A number within a pedigree symbol indicates the number of additional individuals in that generation

Targeted next-generation sequencing was performed alongside histology, immunohistochemistry and microscopic analyses of the brain tissue [10]. A heterozygous missense mutation c.2345G > A (p.Arg782His, rs282860281) was identified in exon 18 of *CSF1R* (Fig. 1) in all three symptomatic siblings (Fig. 2). Other neurodegenerative disease-associated gene mutations were excluded and the mutation was confirmed by genotyping using a custom TaqMan allelic discrimination assay (Life Technologies). The family history is notable for late-onset dementia in the mother at the age of 89 (Fig. 2) and a father who died at age 62 of unrelated causes. Of note, a paternal first cousin once removed may have had symptoms similar to Case #1 (not shown in pedigree). The genetic status of four additional living siblings shown in the pedigree is not provided for privacy reasons. Because of the father’s relatively early death and the fact that neither parent’s DNA was available to test, the penetrance of the mutation cannot be fully assessed.

## Case presentations

All three cases presented with slowed movement, poor balance and cognitive deficits (Table 1). Gait disturbances included retropulsion and postural instability, without tremor, rigidity or cog-wheeling. Early behavioral changes included impulsiveness and disinhibition. MRIs from the three patients were reviewed by several neurologists with expertise in neurodegenerative diseases as well as neuroradiologists. The most consistent finding was increased signal on FLAIR and T2-weighted images in the periventricular and deep white matter; volumetric measurements were not available. Ultimately, the MRI findings were supportive of, but not distinctive for, HDLS, although the MRI pulse sequences were not optimized for the detection of calcifications and other distinctive markers. Case #1, a female in her late 40s, developed depression and difficulty with cognition in addition to motor impairments, and was unable to work after approximately one year. She was noted to be increasingly emotionally labile. Examination at age 52 demonstrated a pseudobulbar affect. Her short-term memory was only mildly impaired, but she exhibited poor emotional regulation, perseveration and a deficit in set-shifting. For example, she was unable to perform the oral version of the Trail Making Test on which she was required to produce an ascending sequence of alternating letters and numbers (e.g., A-1-B-2…). Clinically, she was thought to have frontotemporal degeneration (FTD). Case #2 was a woman in excellent health until age 55. When first evaluated neurologically both mild memory problems and limited use of her left hand were noted. Her personality and behavior were mildly altered in that she was slightly labile emotionally. Examination demonstrated normal cognition except for a moderate impairment in short-term word memory. She had clear but subtle parietal deficits including left visual extinction and a mild left motor neglect manifested as failure to use her left arm unless instructed to do so. There was a mild decrease in left hand dexterity. Her clinical diagnosis was corticobasal syndrome (CBS). Case #3 was a man who presented with symptoms that began in his early 50s. He was found to have poor judgment at work and trouble balancing his checkbook. He became disabled after two years. On examination he exhibited prominent frontal deficits including distractibility, perseveration and mild emotional lability. Memory and visuo-spatial function were poor, with left visual extinction. By MRI, there was thinning of the corpus callosum which was not clearly evident in the other subjects. Clinically, his diagnosis was dementia with Lewy bodies (DLB) and parkinsonism. In all three instances, cognitive/behavioral and motor deficits worsened slowly and relentlessly over several years; subjects were not examined by the authors in their terminal states.

**Table 1:** Clinical characteristics of individuals with the *CSF1R* p.Arg782His mutation

Case	Sex	Origin	Onset age	Age at death	Initial symptoms	Affected family members	Prominent pathology
1^a^	F	USA	late 40’s	56	Cognitive decline, depression, slowed movement	3 siblings	Severe dorsolateral frontal white matter loss, disrupted axons, axonal spheroids
2^a^	F	USA	54	57	Poor balance, cognitive and memory decline, slowed movement	3 siblings	Severe orbital frontal white matter loss, disrupted axons, axonal spheroids
3^a^	M	USA	early 50’s	55	Poor balance, cognitive decline, slowed movement	3 siblings	Severe orbital frontal and parietal white matter myelin loss, disrupted axons, axonal spheroids
4	F	Japan	51	-	Cognitive decline, aphasia, epileptic seizures	3 uncles, cousin	Disrupted axons, axonal spheroids (biopsy)
5	F	USA	51	-	Cognitive and memory decline	3 aunts	Disrupted axons, axonal spheroids (biopsy)
6	F	Korea	37	42	Poor balance, stuttering, dysarthria	sibling, mother, uncle	Frontal and parietal white matter myelin loss, disrupted axons, axonal spheroids

^a^Cases reported in this study

### Neuropathology

**Fig. 3:**
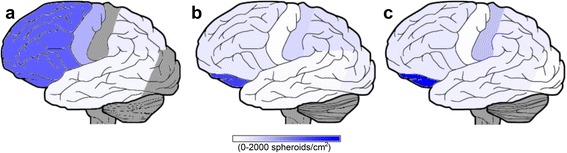
Axonal spheroids were heterogeneously distributed throughout the subcortical white matter across all three subjects. Lateral representations display the relative burden of the subcortical white matter spheroid pathology as density measurements for Cases #1-3 that are mapped to different cortical regions (**a**-**c** respectively). Densities of the APP positive spheroids were obtained from counts and surface areas generated by white matter regions of interest in NIH’s ImageJ 1.49. Dark grey areas indicate regions where densities were not obtained

**Fig. 4:**
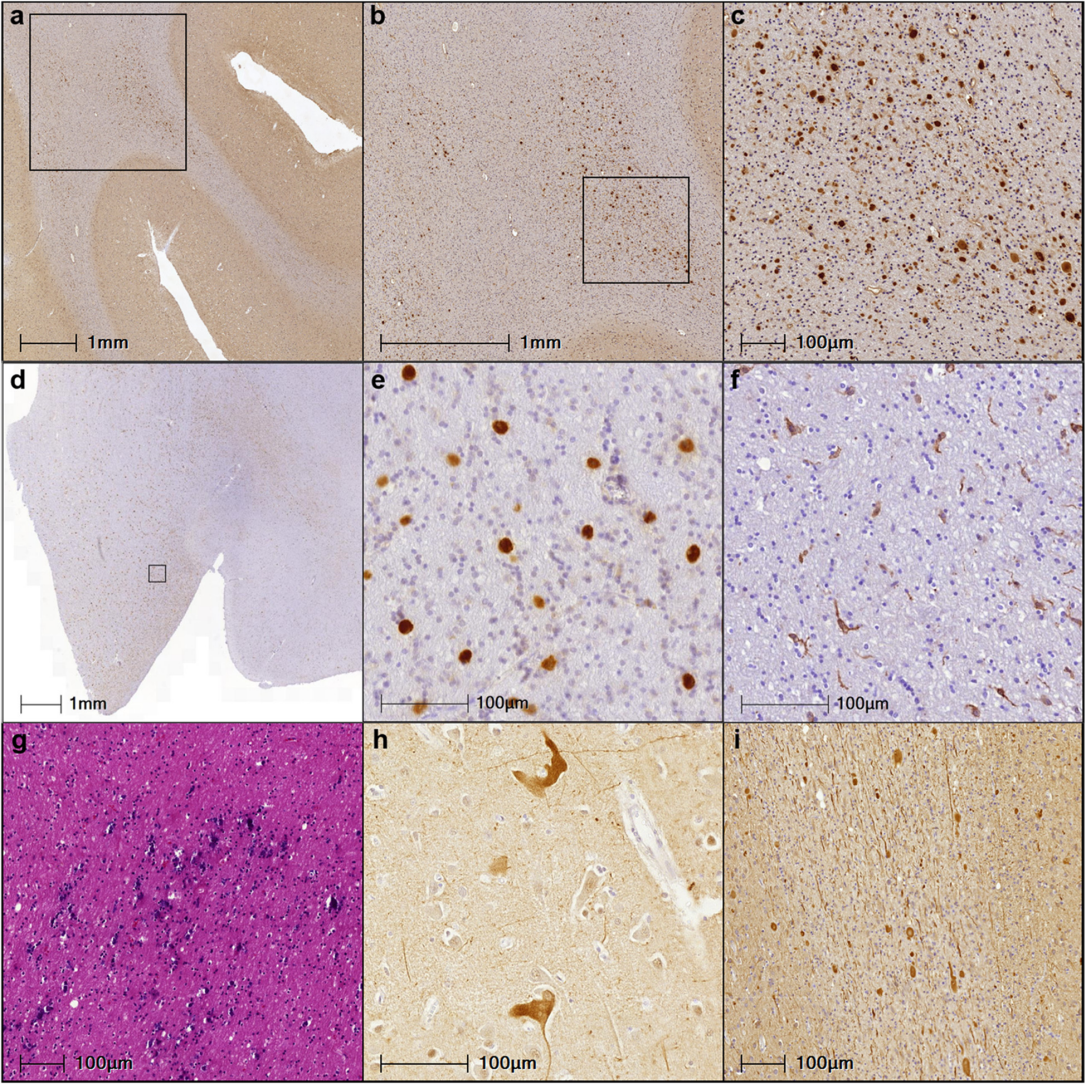
Common neuropathological features of the 3 cases of HDLS studied here. Spheroids were abundant (**a**) primarily in the deeper white matter without affecting the short association fibers; **b** spheroids had a heterogeneous distribution as is evident by comparing the left and right side of the image; **c** focal very dense clusters of spheroids were seen (Case #3, orbital frontal cortex). **d** Microglia were not diffusely distributed and were of two distinct populations. Many microglia had morphologies indicative of (**e**) activated phagocytic macrophages that were CD68 positive and Iba1 negative with unusually ramified morphologies (**f**) while others were Iba1 positive microglia with more simple morphologies (Case #2, anterior corpus callosum). Occasionally, additional pathologies included (**g**) patches of calcifications (Case #2, blue-black splotchy areas in the anterior corpus callosum) and (**h**) irregular neuronal accumulations of pathological tau (Case #3, anterior cingulate). **i** Spheroids could also be visualized by tau, ubiquitin and α-synuclein antibodies (not shown here as well as by anti-neurofilament antibodies (shown here for Case #1 in the angular gyrus). Antibodies and stains used: (a-c) 22c11, (d-e) CD68, (f) Iba1, (g) H&E, (h) PHF1, (i) RMO24.9

Each case showed abundant white matter Aβ precursor protein (APP) positive spheroids that were heterogeneously distributed in subcortical regions (Figs. 3 and 4a-c). These spheroids were also observed by neurofilament (Fig. 4i), tau and ubiquitin antibodies and H&E (not shown). The spheroids were more numerous in deep white matter with the U-fibers relatively spared (Fig. 4a). Phagocytic cells (macrophages and microglia) were also heterogeneously distributed in the subcortical white matter and these cells showed highly varied and distinctly unusual morphologies. The most distinctive morphology was characteristic of activated phagocytic macrophages (Fig. 4d-e) that were strongly positive for CD68, but not Iba1 positive. Other, Iba1 positive microglia had a more classic microglia shape (Fig. 4f). Prominent spongiosis in neocortical regions was another common feature. Neither TDP-43 nor α-synuclein inclusions were observed.

Case #1’s gross examination revealed severe frontal and temporal atrophy with severe ventricular enlargement and a 1092 g brain weight. Microscopically, besides the numerous spheroids (Fig. 3a), additional pathology included obvious neuronal loss and degeneration of long axonal projections, extensive cell loss and gliosis with relative sparing of the granule cells in the hippocampus, rare Aβ plaques and focal cortical amyloid angiopathy (CAA), but no neurofibrillary tangles (NFTs). Case #2 had moderate frontal atrophy with mild ventricular enlargement and weighed 1338 g. There were focal areas of dystrophic calcification and mild demyelination in the corpus callosum (Fig. 4g) and entorhinal cortex that were not apparent in the MRI. Rare ballooned neurons were noted in the cingulate, along with tau-positive grains and neurites in limbic areas, occasional tau-positive coiled bodies, mild CAA without Aβ plaques and NFTs in the medial temporal lobe. Case #3 had mild, diffuse atrophy with mild ventricular enlargement and weighed 1278 g. The limbic regions and brainstem were relatively spared, although spheroids were observed in the medulla. Similar to Case #2, irregular, tau-positive ballooned neurons were also noted in the cingulate (Fig. 4h) while no Aβ plaques were seen but some NFTs were present only in the entorhinal cortex.

## Conclusions

Abundant white matter spheroids and CD68-positive macrophages were the predominant pathologies in these cases. Cases #2 and #3 had rare ballooned neurons, coiled bodies and tau-positive grains and neurites, which are found in other tauopathies such as corticobasal degeneration, progressive supranuclear palsy, Pick’s disease and argyrophilic grain disease [11]. What role pathological tau plays in HDLS has yet to be determined.

The p.Arg782His mutation has been previously reported in three families from Japan (Case #4) [12], USA (Case #5) [13] and Korea (Case #6) [14]. The clinical similarities and differences of our three cases and the additional published cases with the same mutation are highlighted in Table 1. Cognitive difficulties were noted for all cases. Our three patients all presented with slowed movements, as did Case #6 in the Table, but Cases #4 and #5 did not. Postural instability was also common, although this was a late symptom for Case #5. The motor deficits in Cases #4 and #6 were eventually severe, while the others had relatively mild impairments.

CSF1R is a key regulator of myeloid lineage cells and microglia in the adult brain [15] and HDLS-associated *CSF1R* mutations are all located in the protein’s tyrosine kinase domain. Experimental evidence indicates that these mutations cause loss of function [16, 17]. *CSF1R* mutations may also result in haploinsufficiency [18] which, in mice, causes a HDLS-like phenotype [19]. CSF1R’s role as a microglial regulator and the functional deficits associated with *CSF1R* mutations supports the hypothesis that microglia dysfunction may precede the accumulation of axonal spheroids in HDLS. Here we present three familial cases with full neuropathological characterization that demonstrate the range of pathology and clinical phenotypes that can be seen in individuals with the same *CSF1R* mutation. Since the three siblings studied here were diagnosed clinically with FTD, CBS, and DLB, our findings underscore the critical importance of genetic testing for establishing a clinical and pathological diagnosis of HDLS.

## Consent

Informed consent was obtained from next of kin in accordance with institutional review board guidelines of the University of Pennsylvania.

## Abbreviations

HDLS: Hereditary diffuse leukoencephalopathy with spheroids
MRI: Magnetic resonance imaging
*CSF1R*: Colony stimulating factor 1 receptor gene
FTD: Frontotemporal degeneration
CBS: Corticobasal syndrome
DLB: Dementia with Lewy bodies
CAA: Cortical amyloid angiopathy
NFTs: Neurofibrillary tangles
